# Editorial: Epigenetic modulation in cancer

**DOI:** 10.3389/fonc.2026.1867730

**Published:** 2026-05-27

**Authors:** Rossella Tricarico, Priya Mondal, Salvatore Cortellino

**Affiliations:** 1 Department of Biology and Biotechnology “L. Spallanzani”, University of Pavia, Pavia, Italy; 2 Laboratory of Epigenetics, Istituti Clinici Scientifici Maugeri Istituto di Ricovero e Cura a Carattere Scientifico (IRCCS), Pavia, Italy; 3 Department of Biochemistry, Council of Scientific & Industrial Research (CSIR)-Central Food Technological Research Institute, Mysore, India; 4 Academy of Scientific and Innovative Research (AcSIR), Ghaziabad, India; 5 Clinical and Translational Oncology, Scuola Superiore Meridionale, Naples, Italy; 6 Sbarro Health Research Organization (SHRO) Italia Foundation ETS, Turin, Italy

**Keywords:** biomarker discovery, cancer, epigenetic, immunomodulation, tumor microenvironment (TME)

Epigenetic deregulation is increasingly recognized as a central contributor to cancer progression, phenotypic plasticity, immune escape, and therapeutic resistance, with growing implications for biomarker development, patient stratification, and therapeutic intervention (1–3). By shaping gene expression, cell identity, and tumor–microenvironment interactions without altering DNA sequence, epigenetic mechanisms influence clinically relevant tumor phenotypes across multiple cancer types (1–3). This Research Topic brings together five studies that illustrate these functions across complementary settings, including chromatin-based regulation of immune evasion in lung adenocarcinoma, epigenetic stratification of breast cancer, DNA methylation and demethylation dynamics in thyroid and bladder cancer, and small RNA-mediated regulatory networks in non-small cell lung cancer.

Taken together, these contributions show that epigenetic alterations are not simply associated with malignant transformation, but can actively shape disease behavior and therapeutic vulnerability. By spanning chromatin modifiers, DNA methylation and demethylation, and non-coding RNA-mediated regulation, the Topic links mechanistic insight to clinically relevant questions in oncology.

A strong mechanistic contribution is provided by Rao et al., who investigate the role of the histone methyltransferase DOT1L in lung adenocarcinoma. Their study shows that DOT1L promotes immune evasion through H3K79me2-mediated activation of immune checkpoint-related programs, including the JAK1/STAT3/PD-L1 axis. By combining transcriptomic and single-cell analyses with ChIP-seq, pharmacologic inhibition, immune co-culture experiments, *in vivo* metastasis models, and validation in clinical specimens, the authors identify DOT1L as a functional regulator of an immunosuppressive tumor microenvironment. This work is especially relevant to the aims of the Topic because it links a defined epigenetic mechanism to immune suppression and supports the rationale for targeting chromatin regulators to improve antitumor immune responses.

A complementary perspective is offered by Guo et al., who address the clinical value of epigenetic profiling in breast cancer. Using a machine learning-derived epigenetic model (MLEM), the authors define patient subgroups with distinct prognostic features, immune landscapes, and predicted treatment sensitivities. In particular, their results suggest that low-MLEM patients are associated with higher immune infiltration and possible benefit from immunotherapy, whereas high-MLEM patients show poorer prognosis but potential sensitivity to chemotherapy, including vincristine. Rather than focusing on a single pathway, this study illustrates how epigenetic information can be incorporated into clinically oriented stratification models and may support more personalized therapeutic decision-making.

The translational importance of epigenetic regulation is further emphasized in the review by Han et al. on follicular cell-derived thyroid cancer. By discussing DNA methylation, histone modifications, chromatin remodeling, and RNA regulation, the authors show how multiple epigenetic layers converge on tumor progression, dedifferentiation, and treatment resistance. Particularly noteworthy is the discussion of thyroid-specific genes involved in iodine handling, and how their epigenetic dysregulation may contribute to radioiodine refractoriness. The review also underscores the broader idea that epigenetic alterations can serve not only as biomarkers, but also as potentially reversible mechanisms that may be exploited to restore treatment sensitivity.

A related theme is developed by Strasenburg et al., who focus on bladder cancer and on the balance between DNA methylation and active demethylation. Their review is particularly valuable because it highlights not only aberrant methylation patterns, but also the loss of TET-dependent 5-hydroxymethylcytosine as a relevant feature of bladder tumorigenesis. By discussing methylation-based biomarkers, distinct epigenetic trajectories in non-muscle-invasive and muscle-invasive disease, and the therapeutic potential of targeting these pathways, the authors emphasize that methylation and demethylation dynamics may inform diagnosis, prognosis, and treatment. Together with the thyroid cancer review, this contribution reinforces the idea that epigenetic states can influence both tumor identity and therapeutic vulnerability.

The scope of the Topic is broadened further by Song et al., who review the potential role of PIWI-interacting RNAs and PIWI proteins in non-small cell lung cancer (NSCLC). This article extends the epigenetic perspective beyond chromatin modifiers and DNA methylation to include small RNA-mediated regulation. The authors summarize evidence linking the PIWI/piRNA axis to proliferation, metastasis, stemness, chemoresistance, and immune-related therapeutic resistance in NSCLC. In this way, the review highlights an emerging area of cancer epigenetics and suggests that non-coding RNA-mediated regulatory networks may represent additional biomarkers and therapeutic targets in lung cancer.

Taken together, the studies included in this Research Topic support several common conclusions. First, epigenetic deregulation is not merely associated with cancer progression, but can actively drive clinically relevant tumor phenotypes. Second, epigenetic mechanisms operate at multiple levels, from chromatin regulation and DNA methylation to non-coding RNA-mediated control, and influence not only tumor cell-intrinsic programs but also the immune and stromal context. Third, these mechanisms are increasingly relevant to translational oncology, as they may contribute to biomarker discovery, patient stratification, and the identification of new therapeutic opportunities.

At the same time, the Research Topic also highlights the challenges that remain. The effects of epigenetic alterations are often strongly context-dependent, and many proposed biomarkers and therapeutic strategies still require robust biological and clinical validation. Future work will need to clarify which epigenetic changes are functionally central in specific tumor settings and how best to incorporate this knowledge into rational combination therapies and precision oncology approaches (2, 3).

Overall, this Research Topic highlights the growing importance of epigenetic modulation in cancer biology. By connecting mechanistic insight with clinical relevance across multiple tumor types, these studies provide a useful framework for translating epigenetic knowledge into more effective diagnostic and therapeutic strategies (1–4).
